# Laparoscopic common bile duct exploration for retained biliary stent removal after one anastomosis gastric bypass: A case report

**DOI:** 10.1016/j.ijscr.2025.111601

**Published:** 2025-07-04

**Authors:** Jared Levy, Andrei Keidar, Shai Meron Eldar, Adam Abu Abeid

**Affiliations:** a The Faculty of Medical and Health Sciences, Tel Aviv University, Israel; b Division of General Surgery, Tel Aviv Sourasky Medical Center, The Faculty of Medical and Health Sciences, Tel Aviv University, Israel

**Keywords:** OAGB, Common bile duct exploration, Biliary stent, Choledocholithiasis, Case report

## Abstract

**Introduction:**

Cholelithiasis is common following metabolic and bariatric surgery (MBS), with gallstones developing in up to one-third of patients due to rapid weight loss and metabolic changes. Procedures such as Roux-en-Y gastric bypass (RYGB) and one anastomosis gastric bypass (OAGB) alter gastrointestinal anatomy, complicating endoscopic access to the biliary tree and presenting challenges in the management of choledocholithiasis. This report highlights the relationship between MBS and biliary complications, using a case of post-OAGB cholangitis due to a retained stent to illustrate broader management considerations.

**Case presentation:**

A 50-year-old female with a history of vertical banded gastroplasty, laparoscopic cholecystectomy, and prior ERCP with CBD stent placement for choledocholithiasis underwent OAGB. One month postoperatively, she had abdominal pain and fever. An abdominal CT scan showed a retained stent in the CBD. Due to altered anatomy after OAGB, standard endoscopic stent retrieval was not feasible. She underwent laparoscopic CBD exploration with intraoperative ultrasound to identify the retained stent. A longitudinal choledochotomy was performed, the stent and stone debris were removed, and the choledochotomy was closed primarily. Her postoperative course was uneventful.

**Discussion:**

In patients after OAGB, standard biliary access is not feasible, and alternative approaches are required. Laparoscopic CBD exploration is one option that was shown to be effective in this case.

**Conclusion:**

This case highlights the challenge of managing biliary disease in post-MBS patients. A detailed history and file review help prevent stent retention. Laparoscopic CBD exploration offers a safe and effective solution when conventional endoscopic access is limited.

## Highlights

• Management of choledocholithiasis in patients with altered anatomy require a tailored strategy
• Retained stents could be encountered in patients after metabolic and bariatric surgery
• In this case, laparoscopic common bile duct exploration with stent removal was safe and effective

## 1 Introduction

Cholelithiasis is common following metabolic and bariatric surgery (MBS), with studies indicating gallstone formation occurring in approximately 10–38 % of patients [1]. This is related to several mechanisms and risk factors. The primary mechanism is rapid weight loss, which leads to increased cholesterol saturation in bile and promotes gallstone formation [2].

Choledocholithiasis management in patients after MBS may present unique challenges in cases of altered anatomy. Roux en Y gastric bypass (RYGB) and one anastomosis gastric bypass (OAGB) are procedures in which the duodenum is excluded, and this limits access to the ampulla of Vater [3]. As a result, alternative techniques, such as laparoscopic-assisted Endoscopic Retrograde Cholangiopancreatography (ERCP), percutaneous transhepatic cholangiography, or endoscopic ultrasound-guided access, are often necessary and more efficient approaches to access the biliary tree [4].

This study explores the management of choledocholithiasis in patients with altered anatomy following MBS, illustrated by a case of cholangitis caused by a retained biliary stent and stones after OAGB. It highlights the practical challenges in treating biliary disease when endoscopic access is limited and demonstrates how laparoscopic common bile duct (CBD) exploration can be an effective alternative. This work has been reported as being in line with the 2025 SCARE criteria [5].

## 2 Case presentation

We present the case of a 50-year-old female with a history of type 2 diabetes, hypertension, dyslipidemia, and prior vertical banded gastroplasty (VBG) who underwent laparoscopic removal of the VBG ring and cholecystectomy in 2021. One year following cholecystectomy, the patient developed cholangitis and underwent ERCP with papillotomy, stone removal, and placement of a biliary stent in the CBD. The patient was scheduled for elective endoscopic stent removal; however, the patient was lost to follow-up.

In October 2022 (9 months following ERCP), the patient underwent OAGB in another institution due to severe obesity with a body mass index of 38.4 kg/m^2^. The biliary stent was likely overlooked, suggesting the operating surgeons were unaware of its presence at the time of OAGB. One month postoperatively, she presented with epigastric pain radiating to the back, fever, nausea, and vomiting. Laboratory studies showed a WBC count of 12.1 10^3^/μL, C-reactive protein 152 mg/L, bilirubin 0.54 mg/dL, Gamma-glutamyl transferase 135 U/L, and other liver function tests were within normal limits. An abdominal CT scan showed a retained stent in the CBD. She was diagnosed with cholangitis secondary to the retained stent and was initially treated with intravenous antibiotics and fluids.

Due to altered anatomy after OAGB, standard endoscopic stent retrieval was not feasible. The options available for stent removal included laparoscopic CBD exploration or ERCP performance through the excluded stomach. We opted for laparoscopic CBD exploration instead of intraoperative ERCP, as the procedure is relatively straightforward, and our team has the necessary experience and access to a dedicated hepatopancreatobiliary (HPB) service. She was scheduled for elective laparoscopic CBD exploration the next day.

**Fig. 1:**
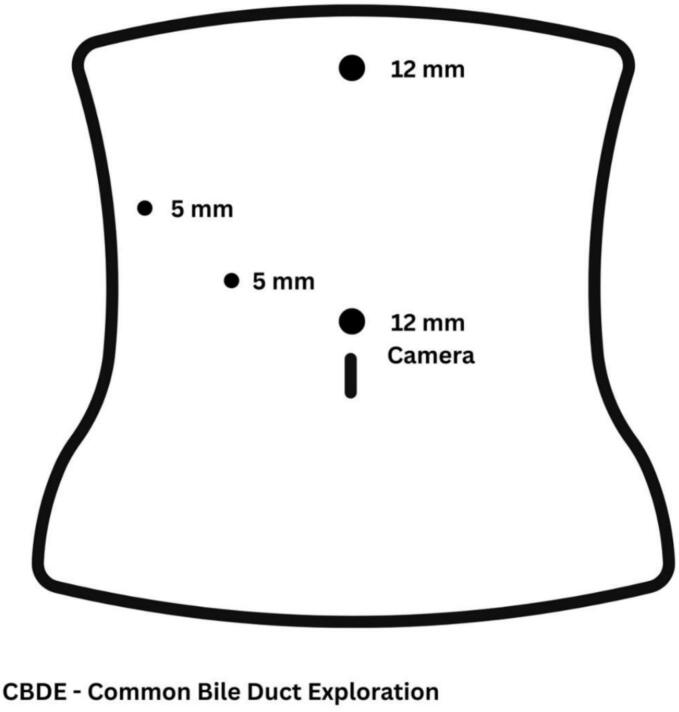
Port placement in Laparoscopic CBDE.

The laparoscopic port positions are illustrated in Fig. 1, and the complete procedure is demonstrated in the accompanying video. The operation began with lysis of omental adhesions from the liver and gallbladder bed. The hepatoduodenal ligament (HDL) was identified and cleared of adipose tissue. The CBD was then visualized along the anterolateral aspect of the HDL and dissected free along its course. Intraoperative ultrasound confirmed the presence of a biliary stent within the CBD. A 3-cm longitudinal choledochotomy was performed. The stent and associated stone debris were completely removed. Intraoperative cholangiography confirmed ductal clearance from stones. The CBD was closed primarily using a single layer of interrupted absorbable sutures (PDS 3–0) and a drain was placed adjacent to the CBD. The operative time was 132 min. The postoperative course was uneventful. The drain was removed on postoperative day 2, and the patient was discharged on postoperative day 3. At the last follow-up (2 years postoperatively), the patient was in good clinical condition with no signs of biliary disease and expressed satisfaction with her recovery and current state of health, despite the prior clinical course.

## 3 Discussion

A patient with a history of cholecystectomy underwent ERCP with biliary stent placement for choledocholithiasis. The patient was lost to follow-up, and the stent was inadvertently left in place. The patient underwent OAGB elsewhere, and following surgery, she suffered from cholangitis, which required surgical removal of the stent via laparoscopic CBD exploration. In this case, the biliary stent was unintentionally retained in the CBD due to loss of follow-up after the index ERCP, which was performed before the patient's OAGB. The patient underwent OAGB at another institution. This case underscores the importance of robust follow-up systems to ensure timely stent retrieval, particularly in patients undergoing OAGB. Preventive measures may include clear documentation in medical summaries, meticulous history taking, and direct communication between physicians. This case reports the clinical and anatomical challenges that can arise when biliary pathology intersects with MBS, particularly in the setting of altered postoperative anatomy.

Several approaches have been developed to access the biliary tree in those with altered gastrointestinal anatomy due to previous MBS. We summarized approaches together with advantages and disadvantages in Table 1. Laparoscopic-assisted ERCP (LAERCP) involves laparoscopic access to the excluded stomach, allowing for a standard ERCP to be performed. This method has high stone clearance rates, ranging from 90 % to 100 %, and is associated with fewer complications than other techniques [6,7]. Balloon-assisted enteroscopy (BAE), using either single- or double-balloon systems, is another option; however, it is technically challenging and has lower success rates - approximately 50 % to 70 % with single-balloon and 63 % to 83 % with double-balloon enteroscopy [6,7]. The ultrasound-directed transgastric ERCP (EDGE) technique involves creating a temporary gastrostomy for endoscopic access. While EDGE boasts a high success rate of up to 97 %, it carries a higher complication risk than LAERCP [7,8]. Percutaneous transhepatic biliary drainage (PTHBD) is an effective but more invasive method for bile duct decompression and stone extraction, particularly in patients where endoscopic options are not feasible [9]. Laparoscopic common bile duct exploration (LCBDE) provides direct surgical access to the CBD and is highly effective, with success rates between 81 % and 100 %. This approach may also be performed concurrently with cholecystectomy when indicated [6,7].

**Table 1:** Comparison of treatment options for choledocholithiasis after gastric bypass.

Approach	Advantages	Disadvantages
Laparoscopic-Assisted ERCP	High success rate (90–100 %), allows use of standard ERCP tools and techniques; facilitates direct access to excluded stomach; enables simultaneous surgical and endoscopic intervention in one setting; widely studied with favorable outcomes in experienced centers [10,11].	Longer operative time (130–184 min); ∼20 % complication rate including pancreatitis, bleeding, and infection; requires full OR setup and close coordination between surgical and endoscopic teams; may not be available in low-resource settings [12].
Balloon-Assisted Enteroscopy	Minimally invasive; avoids surgery and general anesthesia; allows access to biliary tree through natural orifices; may be repeated if needed; suitable for select anatomies [13].	Lower success rates (50–83 %) for bile duct cannulation, technically difficult; challenging anatomy and scope navigation, ∼6.5 % complication rate (perforation, bleeding, pancreatitis) [14,15].
EDGE	High success rate (up to 97 %); avoids laparotomy; provides direct access to the biliary tree via a temporary fistula; can be performed in a staged fashion; emerging as a less invasive alternative to LAERCP [15,16].	∼17 % complication rate (bleeding, stent migration, perforation); ∼10 % risk of persistent gastrogastric or jejunogastric fistula requiring closure; technically complex; requires advanced endoscopic skills and equipment (e.g., Lumen-Apposing Metal Stents (LAMS)); risk of delayed complications [15,16].
PTHBD	Effective for urgent biliary decompression; can be used when endoscopic access fails; performed under imaging guidance; useful in unstable or high-risk surgical patients [17].	Invasive; higher rates of bleeding, bile leakage, and infection; often requires external drainage catheters; not definitive therapy, usually a bridge to surgery or endoscopy; limited availability in some centers [18].
Laparoscopic CBD Exploration	Provides direct visualization and access to CBD; high success rate for complete stone clearance (81–100 %); can be performed with cholecystectomy or as standalone procedure; allows intraoperative cholangiography and stone retrieval [13].	Technically demanding; requires experienced laparoscopic surgeon; risk of bile leak, stricture, or infection; may require T-tube placement or drain; not ideal for patients unfit for general anesthesia [13].

In our case, laparoscopic CBD exploration was chosen to remove the stent. The decision was based on the relative simplicity of the procedure in the experienced hands of a dedicated HPB service at our center. Laparoscopic CBD exploration offers a direct and efficient approach and may be preferable in selected cases when endoscopic access is limited.

## 4 Conclusion

This case illustrates the complexity of managing biliary disease and CBD pathology in patients with altered anatomy following MBS. When standard ERCP is not feasible, interventions such as laparoscopic CBD exploration offer effective alternatives in well-selected cases.

## Informed consent

Written informed consent was obtained from the patient to publish this case and the accompanying images.

## Ethical approval

This study was conducted in accordance with the ethical standards of the institutional and national research committees and the 1964 Helsinki Declaration and its later amendments. Institutional review board (IRB) approval was obtained (IRB No. TLV029524).

## Funding

This study was not supported by any sponsor or funding.

## Author contribution

J.L. wrote the manuscript and prepared the supplemental video. A.K. and S.M.E. revised the manuscript. A.A.A. revised the manuscript and assisted with video preparation. All authors reviewed and approved the final version of the manuscript.

## Guarantor

Jared Levy.

## Research registration number

Not applicable.

## Conflict of interest statement

The authors declare no conflict of interest.

## Data Availability

All data supporting the findings of this case report have been included in this article. No additional datasets were generated or analyzed in the current study.

## Appendix A Supplementary data

Supplementary video Laparoscopic removal of retained CBD stent. Supplementary video
